# TWCOM: an R package for inference of cell–cell communication on spatially resolved transcriptomics data

**DOI:** 10.1093/bioadv/vbae101

**Published:** 2024-07-16

**Authors:** Dongyuan Wu, Susmita Datta

**Affiliations:** Department of Biostatistics, University of Florida, Gainesville, FL 32603, United States; Department of Biostatistics, University of Florida, Gainesville, FL 32603, United States

## Abstract

**Summary:**

The inference of cell–cell communication is important, as it unveils the intricate cellular behaviors at the molecular level, providing crucial insights essential for understanding complex biological processes and informing targeted interventions in various pathological contexts. Here, we present TWCOM, an R package that implements a Tweedie distribution-based model for accurate cell–cell communication inference. Operating under a generalized additive model framework, TWCOM adeptly handles both single-cell resolution and spot-based spatially resolved transcriptomics data, providing a versatile tool for robust biological sample analysis.

**Availability and implementation:**

The R package TWCOM is available at https://github.com/dongyuanwu/TWCOM. Comprehensive documentation is included with the package.

## 1 Introduction

Signaling pathways play an important role in regulating the activities of living organisms, facilitating the transmission of information, response to external perturbations, and the coordination of cellular activities in a highly regulated manner (Hu *et al.* 2021, Hayat *et al.* 2022). At the molecular level, signaling pathways involve a series of events triggered by ligand–receptor (LR) interactions, and these LR interactions occur through cell–cell communications (CCC). Understanding CCC is imperative for unraveling the complexities of cellular behavior and organismal development. To investigate these communications, several computational methods have been developed, particularly through the analysis of single-cell RNA sequencing (scRNA-seq) data (Almet *et al.* 2021, Armingol *et al.* 2021, Wang *et al.* 2023). However, it is crucial to note that scRNA-seq data lack spatial information for individual cells, and since most CCC occur within a limited distance, this limitation may contribute to higher false discovery rates in identifying intercellular communications (Almet *et al.* 2021).

In recent years, the emergence of various spatially resolved transcriptomics (SRT) technologies has brought forth cell physical locations, presenting new opportunities to integrate the spatial distances of cells into CCC analysis. Several methods have been specifically designed for SRT data, including Giotto (Dries *et al.* 2021), SpaOTsc (Cang and Nie 2020), COMMOT (Cang *et al.* 2023), and SpaTalk (Shao *et al.* 2022). In addition to these approaches, CellChat has recently extended its original version developed for scRNA-seq data to incorporate SRT data (Jin *et al.* 2023).

It is important to recognize that two primary types of SRT technologies exist: single-cell resolution technologies, such as seqFISH+ (Eng *et al.* 2019) and STARmap (Wang *et al.* 2018), and spot-based technologies, exemplified by widely used platforms like 10X Visium (Ståhl *et al.* 2016, Salmén *et al.* 2018) and Slide-seqV2 (Stickels *et al.* 2021). While most current SRT-specific CCC approaches treat SRT data solely as single-cell resolution data, acknowledging and incorporating spot-based data is crucial. In spot-based data, each spot location may contain several cells, and even with high-resolution spot-based technologies capable of reaching the size of mammalian cells, cellular overlap within a single spot can occur (Cable *et al.* 2022). Therefore, interpreting the mixture of cell types within one spot for CCC analysis from spot-based data is of significant importance.

To remedy the inadequacies, we developed a novel generalized linear regression model with compound Poisson-Gamma distributions, also known as Tweedie distribution with p∈(1,2), incorporating spot-based data to infer CCC (Wu *et al.* 2023). Notably, this model structure seamlessly accommodates simultaneous communication between different cell types and provides the direction of the association between cell-type communications and LR interactions. Furthermore, our model incorporates a crucial assumption for paracrine or autocrine signaling: the probability of communication diminishes as the distance between cells increases (Francis and Palsson 1997, Doorn *et al.* 2012, Wang *et al.* 2023). In spite of its inferential importance, the previous work BATCOM (Wu *et al.* 2023) was deemed not to be scalable for multiple samples upon our further investigation, due to the computational burden imposed by the Markov chain Monte Carlo sampling framework.

Consequently, we present an R package, TWCOM, designed to execute the novel model structure mentioned earlier and to offer a frequentist inference within the generalized additive model (GAM) framework (Wood 2013, 2017). This package has notably improved the scalability of the model and facilitates user-friendly integration. Simultaneously, we propose specific strategies to address potential limitations.

## 2 Methods

### 2.1 Model structure

The model structure can be expressed as follows:

(1) $$\begin{array}{c} \begin{array}{c} \;\text{log}\;\left(E(C_{i,j}^{k})\right)=\beta_{0}^{k}+\text{exp}(-\rho D_{i,j})\sum_{g_{1},g_{2}}\beta_{g_{1},g_{2}}^{k}M_{i,g_{1}}M_{j,g_{2}}\\ +\nu_{i}^{L}+\nu_{j}^{R},\;i,j=1,2,\ldots ,N. \end{array} \end{array}$$

In this equation, parameters need to be interpreted separately under spot-based and single-cell resolution SRT data settings. For spot-based SRT data, $C_{i,j}^{k}=L_{i}^{k}\times R_{j}^{k}\;(i,j=1,2,\ldots ,N)$ is the communication score from sender cells in a specific spot *i* to receiver cells in a specific spot *j* for LR pair *k*, with *N* being the total number of spots. $D_{i,j}$ is a chosen distance metric between spots *i* and *j*, often considered Euclidean in our applications. Importantly, as the distance $D_{i,j}$ increases, the probability of communication score $C_{i,j}$ decreases. Conversely, for single-cell resolution SRT data, $C_{i,j}^{k}=L_{i}^{k}\times R_{j}^{k}\;(i,j=1,2,\ldots ,N)$ is the communication score from sender cell *i* to receiver cell *j* for LR pair *k*, with $D_{i,j}$ representing the distance between cells *i* and *j*.

The regression coefficients $\beta_{g_{1},g_{2}}^{k}\;(g_{1},g_{2}=1,2,\ldots ,G)$ are the parameters of interest, reflecting the communication strength of LR pair *k* from sender cell type $g_{1}$ to receiver cell type $g_{2}$, where *G* is the total number of cell types. It is important to note that $\beta_{g_{1},g_{2}}^{k}$ is distinct from $\beta_{g_{2},g_{1}}^{k}$. The former corresponds to the coefficient of the interaction between cell type $g_{1}$ from the sender spot or cell *i* and cell type $g_{2}$ from the receiver spot or cell *j*, while the latter represents the coefficient of the interaction between cell type $g_{2}$ from the sender spot or cell *i* and cell type $g_{1}$ from the receiver spot or cell *j*.

The parameter ρ serves as a communication constraint, controlling the rate at which communication decreases with increasing distance. In practice, we will try several different values of the tuning parameter ρ and select the best one based on the Akaike Information Criterion (AIC) (Bozdogan 1987). While this parameter could potentially be estimated using more complicated methods, such as the EM algorithm, we have chosen to select it beforehand and keep it fixed during model fitting. This approach ensures stability and computational efficiency.

In the case of spot-based SRT data, $M_{i,g_{1}}$ and $M_{j,g_{2}}$ represent the proportions of cell types $g_{1}\;(g_{1}=1,2,\ldots ,G)$ at the sender spot *i* and $g_{2}\;(g_{2}=1,2,\ldots ,G)$ at the receiver spot *j*. Conversely, for single-cell resolution SRT data, which is a special instance of spot-based data, one only needs to assign 1 as the cell-type proportion for the specific cell type at the cell *i* or *j*.

Given that $C_{i,j}^{k}$ is not independent, as it partially comes from the same spot or cell, Equation (1) includes two random effects $\nu_{i}^{L}$ and $\nu_{j}^{R}$ to account for the random variation occurring between spots or cells. They are assumed to be independent and identically distributed normal with unspecified variance, which will be estimated when fitting the model.

Since $C_{i,j}^{k}(i,j=1,2,\ldots ,N)$ is expected to be a sparse vector with continuous positive scores in the nonzero positions, we use compound Poisson-Gamma distributions, also known as Tweedie distribution with p∈(1,2), to model it. For a detailed introduction and discussion of the model structure, please refer to Wu *et al.* (2023).

### 2.2 Grid random effects

In Equation (1), it is important to note that we treat the sender spots/cells participating in the signaling from the ligand and the receiver spots/cells involved in the signaling from the receptor as two separate and distinct random effects. This approach results in a total of 2*N* random effect levels. However, a crucial limitation becomes evident—the number of parameters to estimate grows proportionally with the number of random effect levels (Brooks *et al.* 2017). In simpler terms, the computation will become exceedingly slow, especially in scenarios involving high-resolution SRT data or multiple samples of the data.

To address this concern, we mitigate the number of random effect levels by introducing a spatial grid. This process involves partitioning each tissue field into $N_{\text{grid}}$ rectangles, numbered from 1 to $N_{\text{grid}}$. As a result, the sum of the levels of the random effects $\nu_{L}=\nu_{i}^{L}\;(i=1,2,\ldots ,N)$ and $\nu_{R}=\nu_{j}^{R}\;(j=1,2,\ldots ,N)$ is no longer 2*N* but is reduced to $2N_{\text{grid}}$. This strategic reduction enhances computational efficiency without significantly compromising the model’s accuracy, a point we demonstrate in the simulation studies (Supplementary Note 1.1). A biological assumption underpinning this approach is that the gene expression levels of cells within a specific range may correlate with each other. Consequently, the grid random effects can accommodate this assumption to some extent.

### 2.3 Generalized additive model framework

To enhance the model’s scalability, we depart from the Bayesian framework used in BATCOM (Wu *et al.* 2023) and instead adopt the frequentist GAM framework (Wood 2013, 2017) to address CCC problems. In this approach, we refrain from adding any splines to the fixed effects β as defined in Equation (1). This implies that the inference for the fixed effects part remains fundamentally similar to that of the generalized linear model. However, to facilitate smoothness in the random effects, we introduce a ridge penalty, following the methodology elucidated by Wood (2013). More specifically, we regularize the random effects $\nu_{L}$ and $\nu_{R}$ by subtracting individual penalties $\lambda_{L}\nu_{L}^{T}S_{L}\nu_{L}$ and $\lambda_{R}\nu_{R}^{T}S_{R}\nu_{R}$ from the log-likelihood function [as defined in Equation (1)]. Here, the matrices $S_{L}$ and $S_{R}$ are identity matrices, offering control over the penalties of $\nu_{L}$ and $\nu_{R}$, respectively. The degree of penalization is determined by the smoothing parameters $\lambda_{L}$ and $\lambda_{R}$, which are selected using generalized cross-validation (Wood 2017).

## 3 Implementation

**Figure 1.**
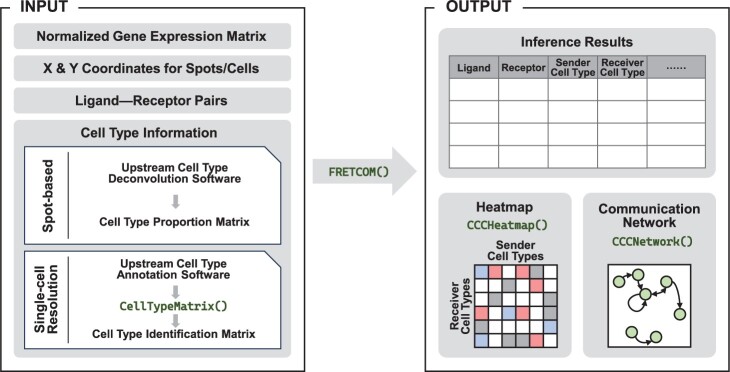
Overview of the TWCOM workflow. TWCOM necessitates four primary inputs: (i) a normalized gene expression matrix derived from SRT data, (ii) X and Y coordinate information for spots or cells, (iii) a list of known LR pairs, and (iv) a matrix depicting the cell-type annotations of spots or cells. Once the model is fitted, users will obtain an inference result table. Furthermore, for visualization purposes, a heatmap and a cell-type communication network can be generated.

TWCOM is coded in the R programming language (≥ v4.3.0) (R Core Team 2023). It is characterized by its user-friendly interface, high flexibility, and ease of use, making it well-suited for inferring cell–cell communication from SRT data, whether at single-cell resolution or spot-based. The workflow of TWCOM is summarized in Fig. 1.

The core function within the TWCOM package is FRETCOM(), named for FREquentist Tweedie modeling for COMmunication. This function is designed to automatically fit the regression model detailed in Section 2.1 and provide frequentist inference. To execute FRETCOM(), users need the normalized gene expression matrix derived from SRT data, a known list of LR pairs [obtainable from public databases, such as CellChatDB (Jin *et al.* 2021), CellPhoneDB (Efremova *et al.* 2020), CellTalkDB (Shao *et al.* 2021)], the X and Y coordinates for each spot/cell in the SRT data, and the corresponding maximum distance permissible for communication. In the case of spot-based SRT data, users must also provide a cell-type proportion matrix for spots, which can be obtained from upstream cell-type deconvolution software tools like RCTD (Cable *et al.* 2022), STRIDE (Sun *et al.* 2022), or SPOTlight (Elosua-Bayes *et al.* 2021). On the other hand, for single-cell resolution SRT data, users simply need to input cell-type information into our function CellTypeMatrix(). This function automatically transforms the information into a cell-type identification matrix usable in the FRETCOM() function.

The output of FRETCOM() includes estimates, *P*-values, and false discovery rate-adjusted *P*-values for each “ligand–receptor–sender cell type–receiver cell type” pair. In addition to the inference for each LR pair, we also provide another function FRETCOMPathway() to infer the CCC for each signaling pathway, which involves multiple LR pairs. For each signaling pathway, we aggregate the communication scores across all LR pairs *k* belonging to the same pathway, i.e. $C_{i,j}=\sum_{k}C_{i,j}^{k}$. Finally, users can utilize CCCHeatmap() and CCCNetwork() to generate a heatmap and communication network of CCC results, respectively, for a specific LR pair or signaling pathway, facilitating visualization.

## 4 Results

### 4.1 Benchmarking

We utilized spot-based data with a compound Poisson-Gamma distribution, previously simulated in Wu *et al.* (2023), to evaluate the performance of our proposed frequentist inference framework of the model (named FRETCOM) in comparison with the prior Bayesian inference framework (named BATCOM). We compared their estimation accuracy and computational efficiency. Further details can be found in Supplementary Note 1.1. In general, FRETCOM demonstrates approximately at least 5 times faster computational speed than BATCOM (Supplementary Table S1), while maintaining good estimation accuracy (Supplementary Figs S1 and S2).

Furthermore, to benchmark our method (FRETCOM) against some state-of-the-art tools, such as Giotto (Dries *et al.* 2021) and COMMOT (Cang *et al.* 2023), we generated synthetic data using SRTsim (Zhu *et al.* 2023). The simulation strategies are elaborated in Supplementary Note 1.2. Supplementary Figure S3 illustrates that FRETCOM exhibits commendable performance based on F1 score and sensitivity when the adjusted *P*-value threshold is ≤.1. Although FRETCOM shows slightly lower specificity and precision compared to COMMOT and Giotto, the differences are not substantial. The KNN version of FRETCOM (${\text{FRETCOM}}_{\text{KNN}}$) performs similarly to the original version (FRETCOM), suggesting that our method effectively handles the assumption of evenly distributed communication among the nearest neighbors.

### 4.2 Case study

To further illustrate the usage of the proposed software, we present a case study using single-cell resolution SRT data obtained from mouse brain tissues specifically designed for investigating Alzheimer’s disease (AD) (Zeng *et al.* 2023). As an example, we analyzed the CCC for signaling pathways from the brain tissues of two 13-month AD mice (disease group) and two 13-month control mice (control group), separately. We only retained cells with at least 100 expressed genes and filtered out genes not expressed in at least 97.5% of the cells. Consequently, the datasets comprised 1777 genes and 25 953 cells, with 14 619 cells from the disease group and 11 334 cells from the control group, categorized into 13 cell types (Supplementary Fig. S4).

**Figure 2.**
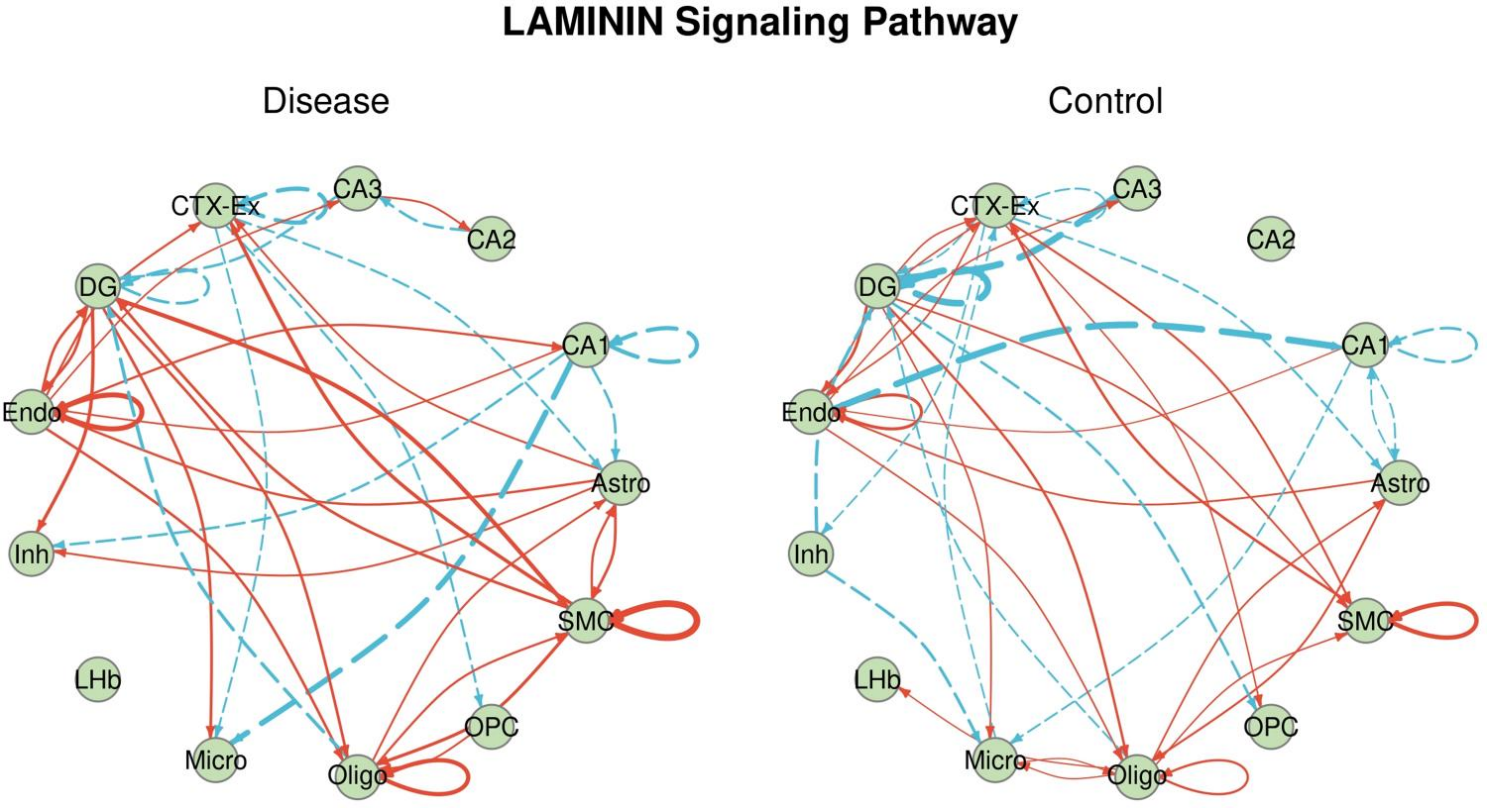
The Inferred intercellular communication networks of the LAMININ signaling pathway for the disease and control groups. A solid edge represents a positive connection between two cell types (i.e. $\beta_{g_{1},g_{2}}>0$), while a dashed edge represents a negative connection between two cell types (i.e. $\beta_{g_{1},g_{2}}<0$). The width of the edge indicates the relative strength of the connection. Astro, Astrocytes; CA1, CA2, CA3, different cellular areas of the hippocampus; CTX-Ex, cortex excitatory neuron; DG, dentate gyrus; Endo, endothelial cell; Inh, inhibitory neuron; LHb, lateral habenula neuron; Micro, Microglia; Oligo, oligodendrocyte; OPC, oligodendrocyte precursor cell; SMC, smooth muscle cell.

Figure 2 presents the inferred CCC networks of the LAMININ signaling pathway for both the disease and control groups. Laminin, known for its strong association with neuronal outgrowth (Luckenbill-Edds 1997), was observed as large punctate deposits in plaques in AD brains (Murtomäki *et al.* 1992, Rodin *et al.* 2020). Previous studies have highlighted a notable increase in the expression levels of cell-type-specific markers for microglia and oligodendrocytes around plaques in AD brains (Zhang *et al.* 2022, Chen *et al.* 2023, Zeng *et al.* 2023). However, in our findings, connections between microglia and oligodendrocytes were absent in the disease group opposed to the control group. Given that oligodendrocytes may release factors stimulating microglia to phagocytose-damaged cells, aiding in debris removal and repair (Peferoen *et al.* 2014), the disappearance of these connections in AD brains may suggest a dysfunction in this mechanism, despite significantly higher expression levels of cell-type-specific markers for microglia and oligodendrocytes in the disease group than the control group.

Additionally, the communication from endothelial cells to hippocampal CA1 neurons shows a remarkable increase compared to the control group to the disease group. It seems that the endothelial cells may release some signals to hippocampal CA1 neurons in the AD brain compared to the control brain, which is aligned with previous discoveries in some contexts, finding that in injured/altered brain endothelial cells secrete factors that are toxic to neurons (Grammas 2000).

## 5 Conclusion

In conclusion, we have introduced TWCOM, an easy-to-use R package specifically designed to construct a generalized linear regression model for the inference of CCC relying on LR interactions. The model incorporates the crucial assumption that the likelihood of communication decreases with increasing distance between cells. Notably, TWCOM is more scalable than BATCOM and other software packages, and also offers a high level of flexibility, enabling efficient inference of CCC for both single-cell resolution SRT data and spot-based SRT data. Additionally, in the case study, we observed some interesting communication or lack of it which is consistent with some earlier scientific facts. We believe that this user-friendly and scalable software would be of much use for identifying CCC using single-cell or spot-based SRT data.

## Supplementary Material

vbae101_Supplementary_Data
