# Supplementary material for: TWCOM: an R package for inference of cell–cell communication on spatially resolved transcriptomics data
Source: Bioinform Adv. 2024 Jul 16;4(1):vbae101. doi: 10.1093/bioadv/vbae101 (PMC11262461; doi:10.1093/bioadv/vbae101)
Supplement: vbae101_Supplementary_Data [file vbae101_supplementary_data.pdf]

# Supplementary materials for TWCOM: an R package for inference of cell-cell communication on spatially resolved transcriptomics data

## Contents

**1 Supplementary Notes** **2**

1.1 Simulation 1: Data Generated from the Compound Poisson-Gamma Model . 2

1.2 Simulation 2: Data Generated from SRTsim . . . . . . . . . . . . . . . . . . . 3

1.3 Case Study . . . . . . . . . . . . . . . . . . . . . . . . . . . . . . . . . . . 5

**2 Supplementary Figures** **7**

**3 Supplementary Tables** **11**

# 1 Supplementary Notes

## 1.1 Simulation 1: Data Generated from the Compound Poisson-Gamma Model

To assess the performance of the proposed cell-cell communication (CCC) approach, we utilized the Compound Poisson-Gamma data previously simulated in Wu et al. (2023) along with the same measurements. We have designated our proposed method as FREquentist Tweedie modeling for COMmunication (FRETCOM). In our assessment, we computed essential metrics, including the true positive rate (TPR), false positive rate (FPR), and observed false discovery rate (FDR). Furthermore, we constructed the receiver operating characteristic (ROC) curve by varying the cutoffs of the adjusted $p$-value to calculate the area under the curve (AUC). For each specific scenario, we determined the mean and standard deviations of these metrics by aggregating results from 100 simulated datasets, thereby providing a comprehensive assessment of the model's performance.

In the simulated data, we generated a panel of 100 spots arranged in a $10 \times 10$ grid. Therefore, we compared three versions of our proposed model: the original model with the $10 \times 10$ grid, resulting in 200 random effects ($\text{FRETCOM}_{\text{original}}$); the model with a new $6 \times 6$ grid, resulting in 72 random effects ($\text{FRETCOM}_{\text{grid}}$); and the null model without any random effect ($\text{FRETCOM}_{\text{nre}}$). Each approach automatically fitted models using $\hat{\rho} = 0.2, 0.5, 0.8$ and then selected the best one based on the Akaike Information Criterion (AIC) (Bozdogan, 1987). Simultaneously, we compared FRETCOM with BATCOM (Wu et al., 2023). BATCOM also fitted models using $\hat{\rho} = 0.2, 0.5, 0.8$ and selected the best one through the Widely Applicable Information Criterion ($\text{WAIC}_2$) (Gelman et al., 2013).

Supplementary Figure 1 depicts the performance of various methods at different true communication constraint parameter values $\rho = 0.2, 0.4, 0.6, 0.8$, while keeping other scenario parameters fixed at $G = 10$, $\phi = 5$, and $p = 1.5$. Notably, the performance of three FRETCOM versions, $\text{FRETCOM}_{\text{original}}$, $\text{FRETCOM}_{\text{grid}}$, and $\text{FRETCOM}_{\text{nre}}$, gradually gets worse as the precision of random effect levels decreases. In general, FRETCOM exhibits a higher TPR compared to BATCOM. However, its FPR and FDR are slightly higher than those of BATCOM. This suggests that the proposed model under the frequentist framework (FRETCOM) tends to be slightly more aggressive than under the Bayesian framework (BATCOM). Despite this, their overall measurement AUC is very similar.

In addition to varying the true communication constraint parameter $\rho$, we also vary other true model parameters $\phi$ and $p$ to assess the performance of different methods (Supplemen-

tary Figure 2). It is worth noting that the fundamental trends among the three versions of FRETCOM and BATCOM remain consistent with those shown in Supplementary Figure 1. Compared to BATCOM, FRETCOM exhibits a more assertive detection for CCC (higher TPR), while still maintaining errors within an acceptable range. Specifically, the FPR and FDR of $\text{FRETCOM}_{\text{original}}$ and $\text{FRETCOM}_{\text{grid}}$ can be controlled to less than 20%.

The primary purpose of proposing the new frequentist implementation framework (FRETCOM) is to enhance the speed, scalability, and usability of the method compared to the Bayesian implementation framework (BATCOM). Therefore, it is crucial to investigate the computational times of each framework. Supplementary Table 1 presents the average computational times in minutes on HiPerGator with one core of an AMD EPYC 75F3 32-Core Processor and 7GB of RAM. We observe that for 100 spots, equivalent to 10,000 observations of the spot-to-spot communication scores in the model, FRETCOM is at least 5 times faster than BATCOM. By reducing the number of random effects (i.e., utilizing the grid version $\text{FRETCOM}_{\text{grid}}$), computational time significantly decreases compared to $\text{FRETCOM}_{\text{original}}$, while maintaining reasonable estimation accuracy, as demonstrated in Supplementary Figures 1 and 2.

## 1.2 Simulation 2: Data Generated from SRTsim

In the second simulation, we employed data generated with the SRTsim simulation design (Zhu et al., 2023) to assess the performance of our models on data simulated from a structure that differs entirely from our proposed model. The single-cell resolution STARmap dataset (Wang et al., 2018) served as the tissue shape for generating gene expression on the tissue. To achieve this, we initially estimated the shape profile based on the spatial locations of the reference data and randomly generated 5000 locations within the estimated shape profile to act as the single-cell locations. We assumed that these single cells belonged to five different cell types and created four equally-sized regions on the tissue, each with distinct cell type compositions. Three cell-type composition scenarios were considered in the simulations (Supplementary Table 2). We identified 930 known ligand-receptor (LR) pairs, comprising 445 ligands and 471 receptors, and randomly assigned 5 out of 25 interactions of cell types for each LR pair. Setting the effect size for LR pairs that mediate the CCC as 5, we simulated the reference-free spatially resolved transcriptomics data with 5000 cells and 2000 genes.

Since this simulation involved 5000 cells, we re-arranged the tissue panel to a more general $10 \times 10$ grid for the random effects $\boldsymbol{\nu}_L$ and $\boldsymbol{\nu}_R$ when utilizing FRETCOM, aiming to reduce the computational burden. We constrained cell communication within $100\mu$m.

However, as SRTsim generated gene expression levels for one cell based on its four nearest neighbors without considering any distance loss (i.e., $\rho = 0$ in our methodology), FRETCOM automatically explored models with four different tuning parameters $\hat{\rho} = 0, 0.2, 0.5, 0.8$ and selected the best one based on the AIC (Bozdogan, 1987). Additionally, we considered a more suited strategy (termed $\text{FRETCOM}_{\text{KNN}}$), directly restricting communications to only occur within the four nearest neighbors of the cells, assuming equal communication activities within these neighbors.

In addition to these two versions of our method, we also compared with Giotto (Dries et al., 2021) and COMMOT (Cang et al., 2023), as they accept simple customized LR pairs. To ensure a fair comparison, all methods examined the same set of 930 known LR pairs mentioned previously. We evaluated the performance of these four CCC methods in detecting the communications from sender cell types to receiver cell types in LR pairs using metrics such as F1 score, sensitivity, specificity, and precision. The assessments were conducted across various adjusted $p$-value thresholds, specifically at 0.001, 0.005, 0.01, 0.05, 0.1, 0.15, and 0.2. To enhance statistical robustness, we carried out 10 simulation replicates for each scenario, calculating both mean and standard deviation to address uncertainties and capture variability effectively.

Supplementary Figure 3 illustrates the benchmarking results of CCC methods using synthetic data from SRTsim. The trends reveal that, with an increase in the adjusted $p$-value threshold, all methods exhibit gradual improvements in sensitivity but experience a decline in specificity and precision. This phenomenon can be likened to the two sides of a coin. However, a comprehensive assessment of overall performance can be obtained by the F1 score, which effectively balances sensitivity and specificity. In general, FRETCOM demonstrates commendable performance when the adjusted $p$-value threshold is less than or equal to 0.1. Notably, in scenario 3, the performance of FRETCOM surpasses other methods. Although FRETCOM exhibits slightly lower specificity and precision compared to COMMOT and Giotto, the differences are not substantial. The KNN version of FRETCOM ($\text{FRETCOM}_{\text{KNN}}$) performs very similarly to the original version (FRETCOM), indicating that our FRETCOM can effectively handle the assumption that communication evenly occurs within some nearest neighbors. It is evident that Giotto displays the worst performance, possibly due to its simplistic statistical approach of insufficiently accounting for the intricate CCC settings.

### 1.3 Case Study

As a case study, we analyzed the CCC for signaling pathways from brain tissues of two 13-month-old AD mice (disease group) and two 13-month-old control mice (control group) separately, using single-cell resolution SRT data (Zeng et al., 2023). We retained cells with at least 100 expressed genes and filtered out genes not expressed in at least 97.5% of the cells. Consequently, the datasets comprised 1,777 genes and 25,953 cells, with 14,619 cells from the disease group and 11,334 cells from the control group, categorized into 13 cell types (Supplementary Figure 4).

We utilized a list of known LR pairs and corresponding signaling pathways from the mouse database in CellChatDB (Jin et al., 2021). After filtering out signaling pathways lacking information within the dataset, we considered 17 signaling pathways for the disease group and 12 signaling pathways for the control group. Regarding cell type interactions, we filtered out some interactions due to minimal or non-existent observations, resulting in 89 interactions (out of 169) for the disease group and 87 interactions (out of 169) for the control group.

Since this real data is single-cell resolution data sequenced using STARmap (Wang et al., 2018), we set the maximum communication distance as $200\mu$m and tried two different values of the tuning parameter $\rho$ as $\hat{\rho} = 0.03$ and 0.05. The decreasing trend of the exponential term $\exp(-\rho D)$ in Equation (1) is shown in Supplementary Figure 5. These two values appeared to be reasonable choices, ensuring a moderate decreasing rate of the communication probability.

# References

H. Bozdogan. Model selection and akaike's information criterion (aic): The general theory and its analytical extensions. *Psychometrika*, 52(3):345–370, 1987.

Z. Cang, Y. Zhao, A. A. Almet, A. Stabell, R. Ramos, M. V. Plikus, S. X. Atwood, and Q. Nie. Screening cell–cell communication in spatial transcriptomics via collective optimal transport. *Nature Methods*, pages 1–11, 2023.

R. Dries, Q. Zhu, R. Dong, C.-H. L. Eng, H. Li, K. Liu, Y. Fu, T. Zhao, A. Sarkar, F. Bao, et al. Giotto: a toolbox for integrative analysis and visualization of spatial expression data. *Genome biology*, 22:1–31, 2021.

A. Gelman, J. Carlin, H. Stern, D. Dunson, A. Vehtari, and D. Rubin. *Bayesian Data Analysis (3rd ed.)*. Chapman and Hall/CRC, 2013.

S. Jin, C. F. Guerrero-Juarez, L. Zhang, I. Chang, R. Ramos, C.-H. Kuan, P. Myung, M. V. Plikus, and Q. Nie. Inference and analysis of cell-cell communication using cellchat. *Nature communications*, 12(1):1088, 2021.

X. Wang, W. E. Allen, M. A. Wright, E. L. Sylwestrak, N. Samusik, S. Vesuna, K. Evans, C. Liu, C. Ramakrishnan, J. Liu, et al. Three-dimensional intact-tissue sequencing of single-cell transcriptional states. *Science*, 361(6400):eaat5691, 2018.

D. Wu, J. T. Gaskins, M. Sekula, and S. Datta. Inferring cell–cell communications from spatially resolved transcriptomics data using a bayesian tweedie model. *Genes*, 14(7): 1368, 2023.

H. Zeng, J. Huang, H. Zhou, W. J. Meilandt, B. Dejanovic, Y. Zhou, C. J. Bohlen, S.-H. Lee, J. Ren, A. Liu, et al. Integrative in situ mapping of single-cell transcriptional states and tissue histopathology in a mouse model of alzheimer's disease. *Nature Neuroscience*, 26(3):430–446, 2023.

J. Zhu, L. Shang, and X. Zhou. Srtsim: spatial pattern preserving simulations for spatially resolved transcriptomics. *Genome Biology*, 24(1):39, 2023.

# 2 Supplementary Figures

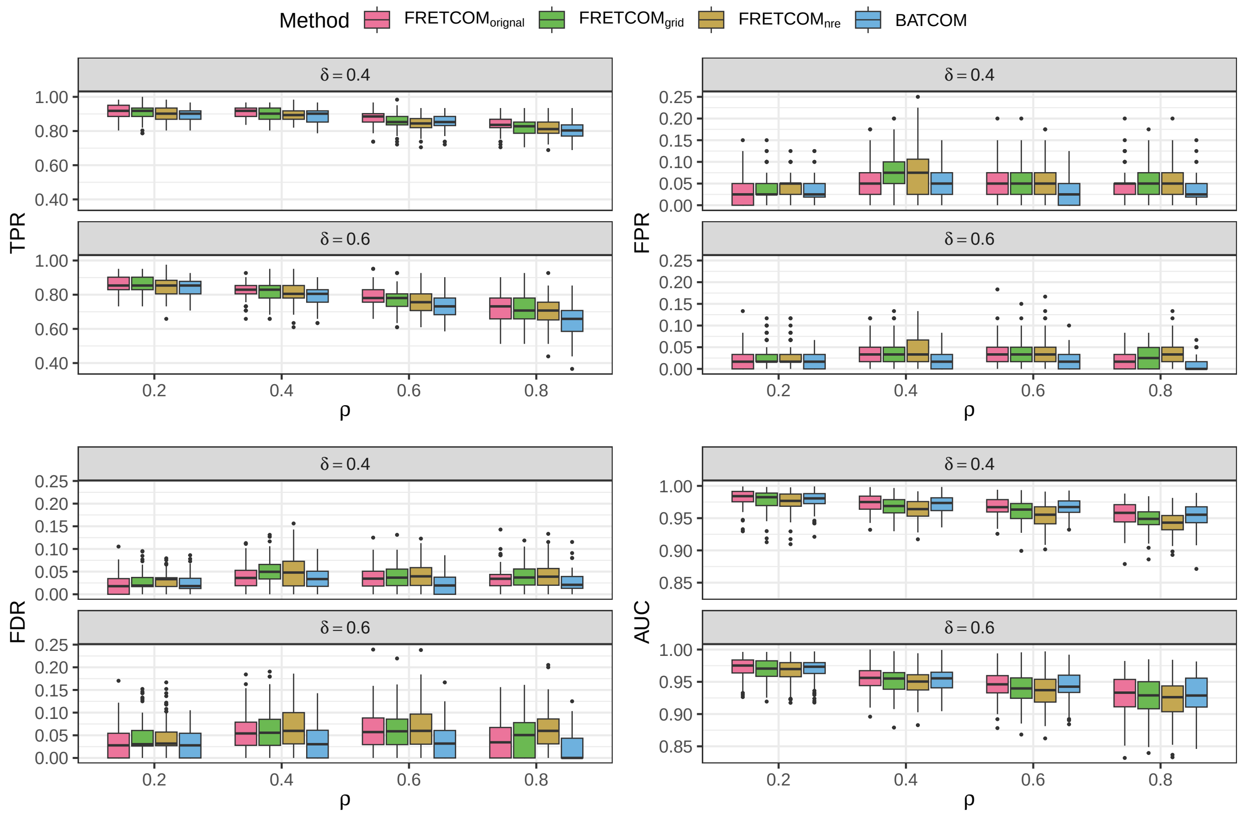


Supplementary Figure 1: Results of different methods when the true communication constraint parameter $\rho$ takes values of 0.2, 0.4, 0.6, and 0.8, based on simulation data generated from the proposed compound Poisson-Gamma model. All scenarios were $G = 10$, $\phi = 5$, and $p = 1.5$. TPR: true positive rate; FPR: false positive rate; FDR: false discovery rate; AUC: area under the ROC curve.

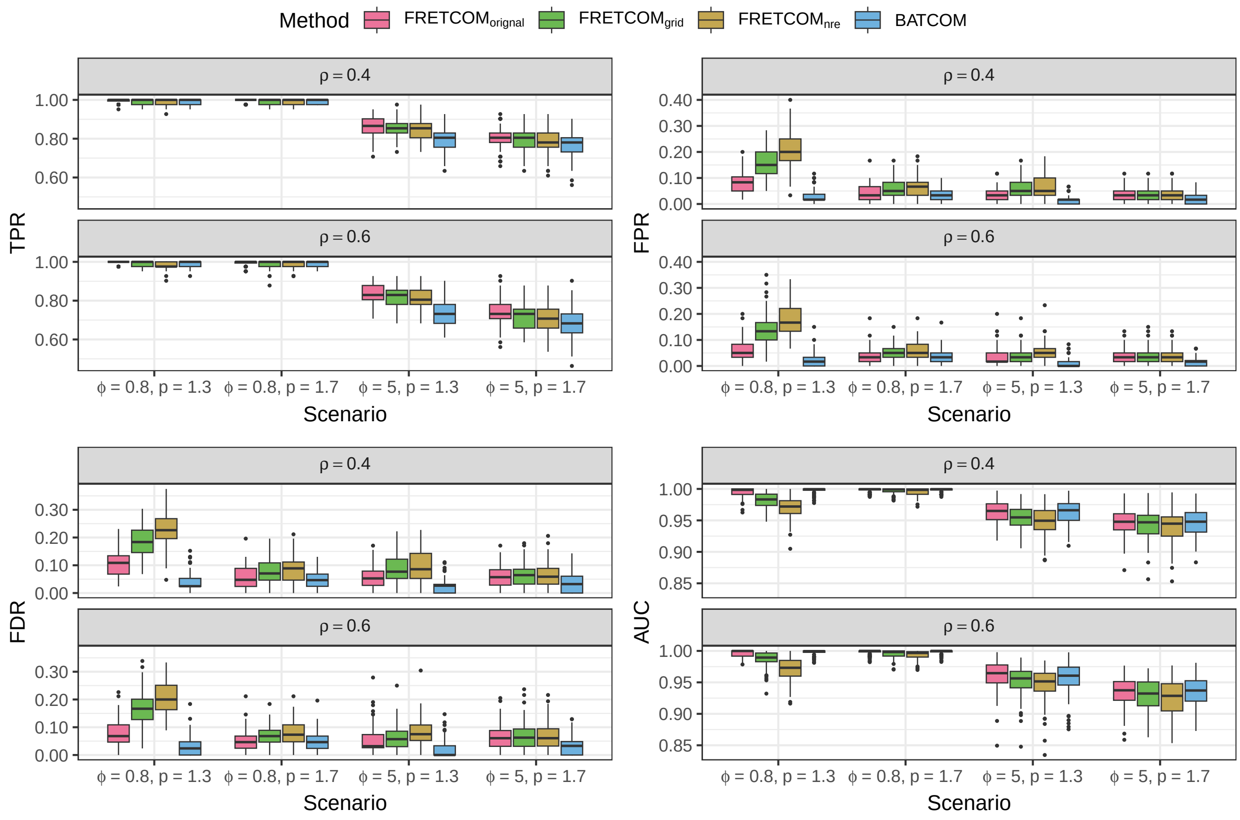


Supplementary Figure 2: Results of different methods based on the simulation data generated from the proposed compound Poisson-Gamma model. All scenarios were $G = 10$ and $\delta = 0.6$. TPR: true positive rate; FPR: false positive rate; FDR: false discovery rate; AUC: area under the ROC curve.

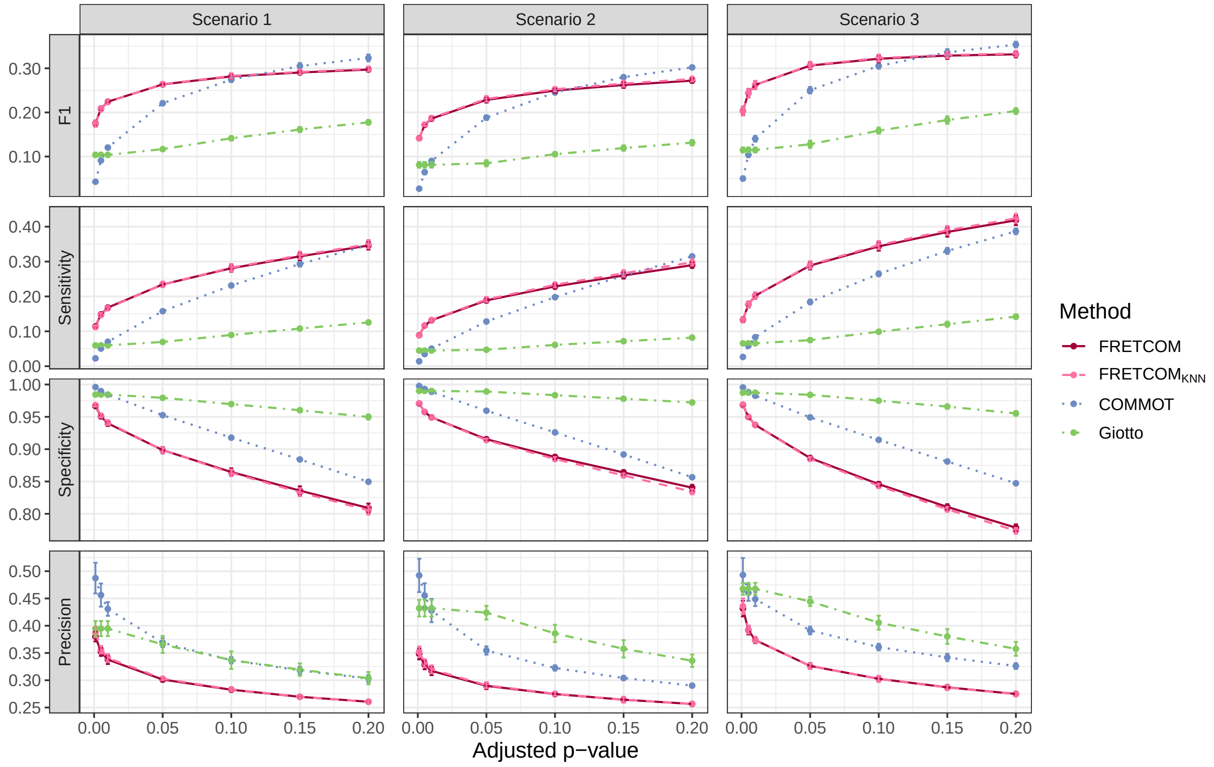


Supplementary Figure 3: Benchmarking results of CCC methods using synthetic data from SRTsim.

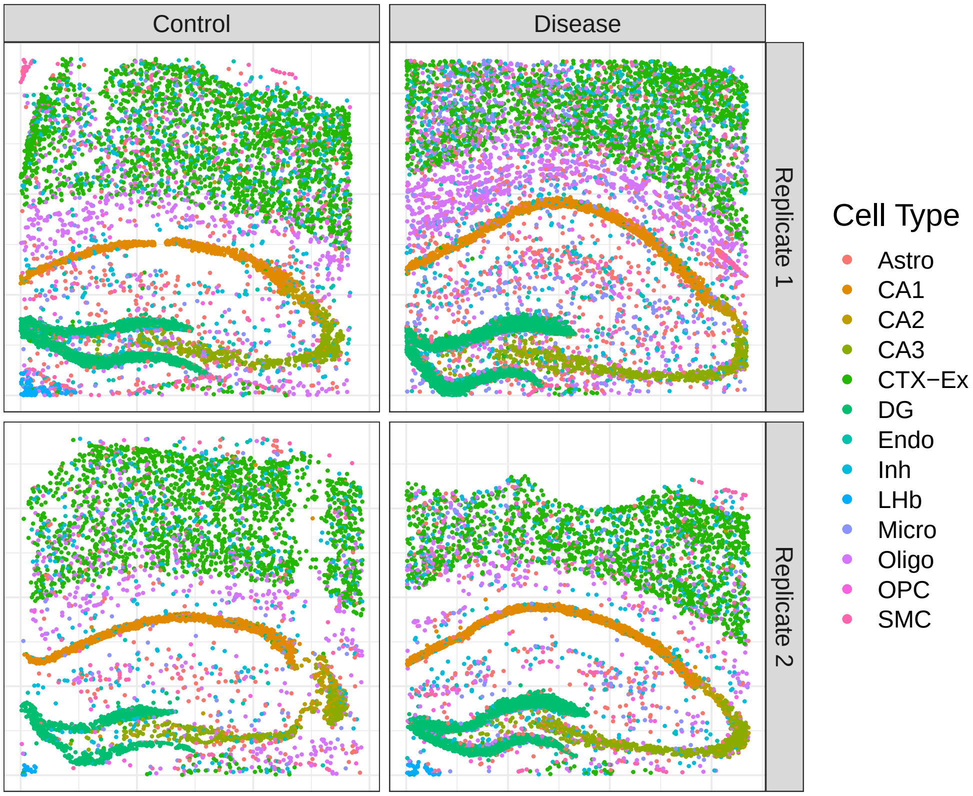


Supplementary Figure 4: Spatial atlas of top-level cell types of samples in the disease and control groups. Astro: Astrocytes; CA1, CA2, CA3: Different cellular areas of the hippocampus; CTX-Ex: Cortex excitatory neuron; DG: Dentate gyrus; Endo: Endothelial cell; Inh: Inhibitory neuron; LHb: Lateral habenula neuron; Micro: Microglia; Oligo: Oligodendrocyte; OPC: Oligodendrocyte precursor cell; SMC: Smooth muscle cell.

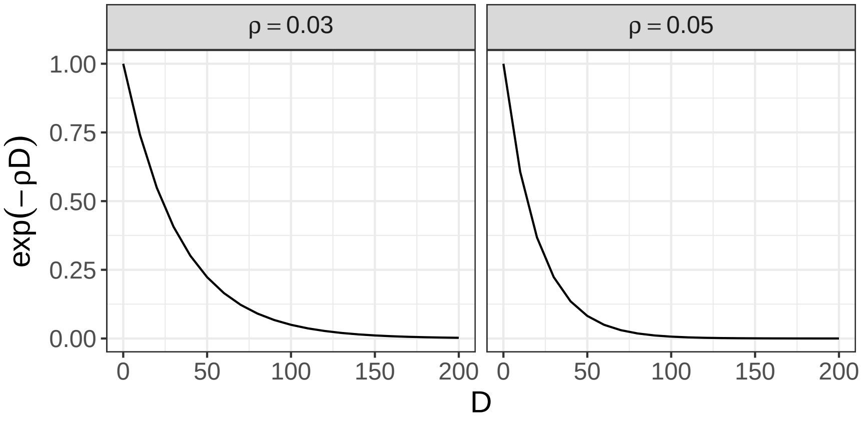


Supplementary Figure 5: The decreasing rate of $\exp(-\rho D)$ as $D$ increases. $D$ represents the distance between two cells/spots, and $\rho$ is the communication constraint tuning parameter.

# 3 Supplementary Tables

Supplementary Table 1: Computational times in minutes of different methods in simulation study 1.

| Method | 5 cell types | 10 cell types | 15 cell types |
|---|---|---|---|
| $\text{FRETCOM}_{\text{original}}$ | 0.55 | 1.38 | 3.87 |
| $\text{FRETCOM}_{\text{grid}}$ | 0.20 | 0.49 | 1.79 |
| $\text{FRETCOM}_{\text{nre}}$ | 0.09 | 0.22 | 0.69 |
| BATCOM | 5.23 | 7.54 | 16.40 |

Supplementary Table 2: Simulation settings for cell-type composition scenarios using SRT-sim.

| | Cell type 1 | Cell type 2 | Cell type 3 | Cell type 4 | Cell type 5 |
|---|---|---|---|---|---|
| | | | *Scenario 1* | | |
| Region 1 | 55% | 30% | 5% | 5% | 5% |
| Region 2 | 5% | 55% | 30% | 5% | 5% |
| Region 3 | 5% | 5% | 55% | 30% | 5% |
| Region 4 | 5% | 5% | 5% | 55% | 30% |
| | | | *Scenario 2* | | |
| Region 1 | 40% | 25% | 25% | 5% | 5% |
| Region 2 | 5% | 40% | 25% | 25% | 5% |
| Region 3 | 5% | 5% | 40% | 25% | 25% |
| Region 4 | 25% | 5% | 5% | 40% | 25% |
| | | | *Scenario 3* | | |
| Region 1 | 70% | 5% | 5% | 5% | 15% |
| Region 2 | 5% | 70% | 5% | 5% | 15% |
| Region 3 | 5% | 5% | 70% | 5% | 15% |
| Region 4 | 5% | 5% | 5% | 70% | 15% |
